# Spin Currents Induced in Open-Shell Molecules by Static and Uniform Magnetic and Electric Fields in the Presence of a Spin–Orbit Coupling Interaction and Conservation Law

**DOI:** 10.1021/acs.jctc.3c00017

**Published:** 2023-04-27

**Authors:** Francesco Ferdinando Summa

**Affiliations:** Dipartimento di Chimica e Biologia “A. Zambelli”, Università degli Studi di Salerno, via Giovanni Paolo II 132, Fisciano 84084, SA Italy

## Abstract

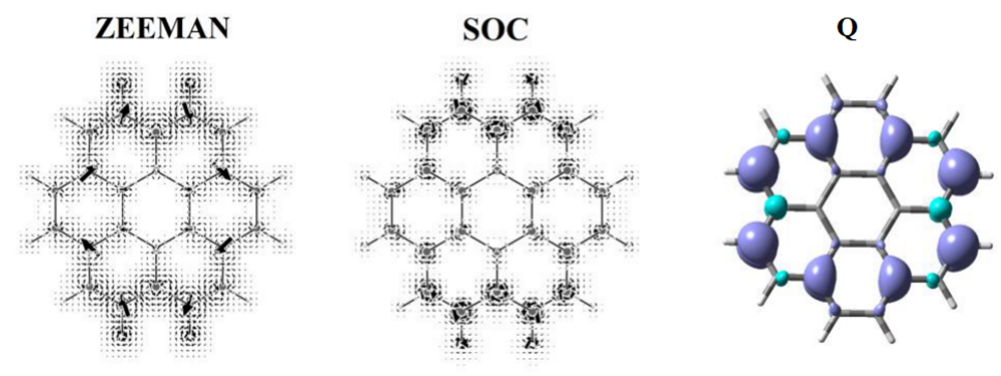

The derivation of the total induced current density vector field, in the presence of static and uniform magnetic and electric fields, is illustrated in a more clear and formally correct language together with a discussion on the charge-current conservation law not presented before for the spin–orbit coupling contribution. The theory here exposed turns out to be in fully agreement with the theory of Special Relativity and it is applicable to open-shell molecules in the presence of a nonvanishing spin orbit coupling. The discussion here exposed turns out to be accurately valid for a strictly central field due to the chosen approximation of the spin–orbit coupling Hamiltonian, but it is appropriate to deal correctly with molecular systems. The ab initio calculation of spin current densities has been implemented at both unrestricted Hartree–Fock and unrestricted DFT levels of theory. Some maps of spin currents on molecules of interest, i.e., the CH_3_ radical and the superoctazethrene molecule are also illustrated.

## 1 Introduction

The interaction of matter with electric and magnetic fields or both has always fascinated the scientific community. Particularly in the case of open shell systems, NMR and EPR spectroscopies^1−3^ are useful means to identify their structure being sometimes the existence of these species very short. However, the aim of the present paper is not to discuss about these spectroscopies but rather to analyze in detail the expressions obtained for the current density vector field induced by static and uniform magnetic and electric fields when the interaction between electrons spins and the applied fields is properly taken into account using the Foldy–Wouthuysen diagonalization of the Dirac Hamiltonian.^4^ A magnetic field induces current distributions in the electron cloud of a molecule. Some of these current distributions can be derived starting from the continuity equation, using the Schrödinger equation with an hydrodynamical approach,^5^ but contributions that arises from electron spin cannot be obtained in this way (being the spin operator self-adjoint) and different procedures have been proposed during the years to overcome this problem.^2,3,6−9^ Of these procedures we recall in particular the Gordon decomposition of the Dirac four-current^6,10−12^ that introduces a magnetization current, see eq 38 for definition, starting from a correct, relativistic spin 1/2 theory. However, this decomposition does not allow to obtain the spin–orbit coupling current derived first by Hodge and co-workers for the hydrogen atom^9^ adopting the Landau approach^7^ and then extended to deal with many electron systems.^13^ The aim of the present paper is to discuss about the continuity equation related to the total induced current density in the presence of a non vanishing one electron spin–orbit coupling interaction in open-shell systems.

## 2 Outline of Notation and Theoretical Methods

Within the Born–Oppenheimer (BO) approximation,^14^ for a molecule with *n* electrons and *N* clamped nuclei, charge, mass, position, canonical and angular momentum of the *k*-th electron are indicated, in the configuration space, by – *e*, *m*_e_, ***r***_*k*_, , , *k* = 1, 2, ..., *n*, using boldface letters for electronic vector operators. Analogous quantities for nuclei are *Z*_*a*_*e*, *M*_*a*_, ***R***_*a*_, etc., for *a* = 1, 2, ..., *N*. Throughout this work, SI units are used and standard tensor formalism is employed, e.g., the Einstein convention of implicit summation over two repeated Greek indices is applied. The third-rank Levi–Civita skew-tensor is indicated by ϵ_*αβγ*_. The imaginary unit is represented by a Roman i. Let us introduce the general definition of *n*-electron probability density matrix functions for a stationary state wave function Ψ(***X***)
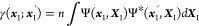
1of electronic space-spin coordinates ***x***_*k*_ = ***r***_*k*_ ⊗ η_*k*_, *k* = 1, 2, ..., *n*, where
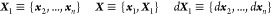
2

By integrating over the spin variable η_1_, a spatial probability density matrix is obtained
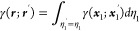
3

Similarly, the spin-density matrix is defined as
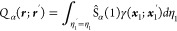
4with  the α component of the spin operator equal to
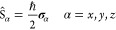
5and **σ**_α_ the Pauli matrices
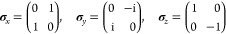
6

The diagonal elements of the density matrix, eq 1, γ(***r***) = γ(***r***; ***r***), give the electronic charge distribution of the state ρ(***r***) = −*eγ*(***r***), and the diagonal elements of eq 4 give the spin density, described by the axial vector *Q*_α_(***r***) = *Q*_α_(***r***; ***r***). For the reference state Ψ_*a*_ the probability density becomes
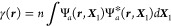
7

Our starting point is the generalized Foldy–Wouthuysen Hamiltonian,^4^ within the Breit–Pauli picture, in the Born–Oppenheimer approximation,^14^ for molecular interacting systems with static and uniform magnetic and electric fields, i.e.,^4,8,13,15^
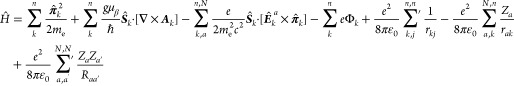
8

In the previous equation primes mean that, performing the double summation, *k* ≠ *j* and *a* ≠ *a*′, μ_*B*_ is the Bohr magneton, *g* is the electron spin g-factor,  is the electric field operator defined as
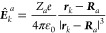
9and  is the electron mechanical momentum operator defined to be
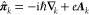
10

It is worth noticing that in Hamiltonian (eq 8) we have removed the electron rest mass energy, so that the zero of our energy axis agrees with the conventional nonrelativistic one. Other high order terms have been not taken into account. The reason to avoid such terms, like the mass-velocity correction, as defined in the Cowan–Griffin Hamiltonian,^16^ is that it makes the Hamiltonian unbounded from below,^17,18^ indeed it converges only for , whereas the spectrum of operator  is (0, + *∞*).^19^ The leading relativistic corrections are futile because it is well-known that the expectation values , *n* ≥ 5, diverge for S states of the hydrogen atom and, in general, for the ground state of any atomic or molecular system.^20−22^ In order to take into account these or other effects it is preferable to use a fully relativistic 4-components approach being in principle simpler to apply. An important aspect of the discussion that will follow is that spin currents as derived in the present manuscript can be implemented both in a traditional (non perturbed) open-shell Hartree–Fock and open-shell DFT calculation because contributions as the spin–orbit coupling and the electron spin Zeeman Hamiltonians can be neglected in a linear response approach calculation at first insight. The implementation, here reported, seems to be extendable in a current density functional theory picture too.^23,24^

## 3 Semi-Relativistic Quantum Mechanical Current Density

To show how the QM expression for the total many body current density ***J*** can be obtained, the Landau–Lifshitz approach^7^ is used which is based on relation
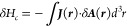
11

To use this idea for a QM system, Landau and Lifshitz argued that the classical Hamiltonian *H*_*c*_ is to be identified with the expectation value of the QM Hamiltonian according to
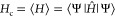
12

Due to eq 11, only terms containing the vector potential ***A*** (or the magnetic field ***B***) must be taken into account. For a complete derivation of the Landau–Lifshitz approach, see ref (9). Looking at the interaction Hamiltonian 8, one can see that the vector potential appears only in three terms. Then, defining
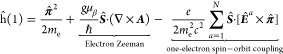
13and using eq 1 we can focus only on the term^8^
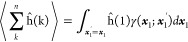
14

The use of the reduced Hamiltonian 13 and the reason to neglect contributions (such as terms like, for example, Fermi contact, spin dipolar interaction, Darwin, and all the others not written in eq 8, see refs (8, 25) for a complete description), except the two-electron spin–orbit coupling term, is that they make no direct contribution to the induced electronic current density vector field in a finite field approach. That is not true in case of a perturbative approach.^2,26^ Furthermore, being in the Born–Oppenheimer approximation, it is clear that the nuclear Zeeman interaction and the term representing the nuclear magnetic moment acting on the moving electrons^25^ cannot be taken into account to derive an expression for the approximate total induced current density being nuclei clamped in space. The reason why the two-electron spin–orbit coupling contribution has been neglected in Hamiltonian 8 is that the author wants to keep as simple as possible the derivations and the discussion carried out in the present manuscript, being in line with the original paper by Foldy and Wouthuysen.^4^ The use of this approximate form of the spin–orbit operator turns out to be accurately valid for a strictly central field.^25^ Despite this, that is not exactly true in the case of molecular systems (as it is the case with atomic systems), the conclusions reported at the end of the present manuscript seem to fully reflect those obtained using a fully relativistic 4-components approach in molecules where Special Relativity plays a fundamental role at least at qualitative level.^27^ For this reason the model here proposed seems to be appropriate to deal correctly with molecular systems. When spin-effects are not taken into account in , it is possible to complete the integration over spin variable and write the following equation
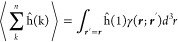
15recovering the spinless density (eq 3) introduced before. This is the case of the first term in eq 13. To obtain the current density vector ***J*** from eq 11, we need to calculate the variation *δH*_*c*_ with respect to an infinitesimal change of the vector potential^9,13^
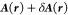
16

Compared to ref (13), a more clear and formally correct derivation of the many-body induced current density will be given here. First, let us consider the term . By expanding  we have
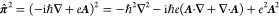
17

Substituting this expression and the definition 3 in eq 15, we can write
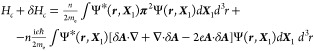
18because
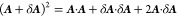
19and a second order variation δ***A***·δ***A*** is not considered being vanishing small at first-order approximation. From the previous equations, it follows that
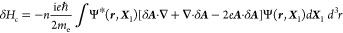
20Let us consider now the term  · (δ***A***Ψ). If we apply the vector identity
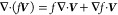
21with *f* = Ψ and ***V*** = δ***A***, we obtain
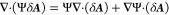
22Multiplying the last equation on the left by Ψ*, one can write
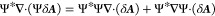
23

The term Ψ*Ψ·(δ***A***), on the r.h.s of the previous equation, can be identified with *f*·***V***, in which *f* = Ψ*Ψ and ***V*** = δ***A***, so using again the identity 21, one obtains
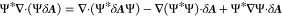
24Considering that
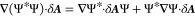
25eq 23 can be rearranged in the form
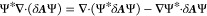
26Applying the divergence theorem for the first term on the r.h.s of the last identity and considering that the wave function goes to zero at infinity, we have
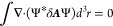
27from which relation 20 can be rewritten as
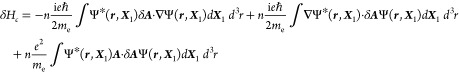
28Comparing this last equation with eq 11, it follows that the contribution given from the first term in eq 13 to the total induced current density vector is
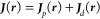
29conventionally rewritten as
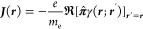
30with
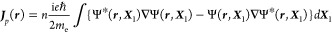
31
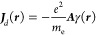
32

A similar procedure can be applied to the spin Zeeman and to the one-electron spin orbit coupling Hamiltonians. Considering the spin Zeeman Hamiltonian first and substituting ***A***(***r***) with eq 16, as before, we obtain that
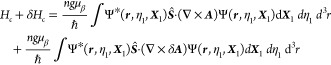
33from which it is possible to rewrite
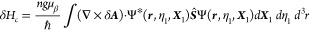
34

Now taking into account the vector identity
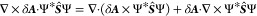
35and applying the divergence theorem for the first term on the r.h.s of the previous equation, considering that the wave function goes to zero at infinity, we have
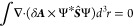
36from which relation 34 can be rewritten as
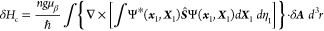
37

Using eqs 11 and 4, we obtain from the Spin Zeeman Hamiltonian as contribution to the total induced current density vector the expression
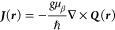
38called also magnetization current in analogy with the one obtained in classical electrodynamics.^9,28^ Now, let us use relation 14 for the last term of eq 13, i.e. the one-electron spin–orbit coupling Hamiltonian, substituting ***A***(***r***) with eq 16, we get
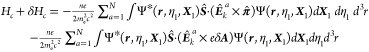
39from which it follows
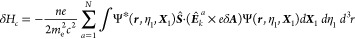
40Using the vector identity
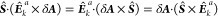
41we obtain
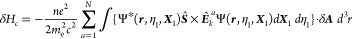
42that using eq 11 enable use to achieve as contribution to the total current density
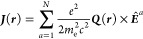
43

As approximation to the total induced many-body current density,^13^ for a generic open-shell system in the Born–Oppenheimer approximation and in the case of a strictly central field, (the two electron spin–orbit coupling interaction has been not taken into account) by collecting all the previous terms, it is possible to write
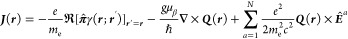
44

The presence of other terms usually included in the Hamiltonian, like the Darwin correction for example or the Coulomb interaction between charged particle, (i.e., terms without the vector potential ***A***), do not change the results that have been illustrated in eq 44 as discussed before. The current density defined in eq 44 is by definition gauge-invariant only for an exact calculation or in the limit of a complete basis set. This gauge dependence comes from the nonrelativistic part of the current density, see eq 30.^29^ In the SI system, units of ***J*** are . As stated before, the results here proposed are to be taken into account only in the presence of static and uniform magnetic and electric fields, so dynamic currents are not considered.^30−35^ The Hamiltonian 13 describes the interaction with the intramolecular perturbation, that is, the intrinsic magnetic dipoles **μ**_*a*_ = γ_*a*_***I***_*a*_, expressed via the magnetogyric ratio  and spin , with *n*_*a*_ an integer, of nucleus *a* via the vector potential , and with an external, spatially uniform and time-independent magnetic field 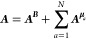
45
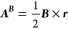
46
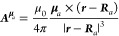
47

Due to the form of the vector potential here chosen, a classical Larmor contribution coming from the nuclear magnetic dipole is also expected, indeed^26^eq 32 can be rewritten as
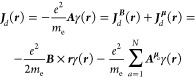
48Here, according to definition 4, it is clear that
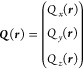
49

In accordance with the Wigner–Eckart theorem, the spin densities are all the same except for a proportionality constant,^3,8,25^ so it is therefore expedient to introduce a reduced spin density scalar function common to all components of the multiplet
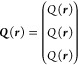
50with
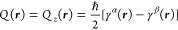
51where γ^α^(***r***) and γ^β^(***r***) represent the densities of spin-up and spin-down electrons, respectively. Recently a topological analysis of the spin density has been reported in literature.^36^ For an open shell molecule, as we can see from the previous equations, we have many spin–orbit coupling current densities as the number of atoms contained, but due to the presence of , these currents are negligible except for very heavy atoms where spin–orbit coupling plays a fundamental role or in systems with high spin multiplicity in the nuclear proximity.^13^ The eq 44 for a closed shell system reduces to
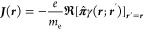
52

The continuity equation associated with the total current defined in eq 44 that is verified, in tensorial notation, is
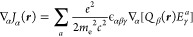
53because the magnetization current has no divergence due to the presence of the curl in its definition, see eq 38, and the non relativistic current (eq 30) can also be obtained with an approach that adopts the Coulomb gauge. That ensures that its divergence is zero for an exact calculation. Now, let us consider the term defined in eq 53. If we use the vectorial relation
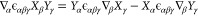
54given for two generic vectors ***X*** and ***Y***, we have that
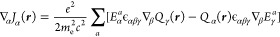
55

Now the second term can be evaluated using the following Maxwell’s equation^37^
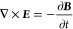
56in the case of a static magnetic field to be
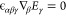
57so we have
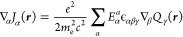
58

Due to the scalar product between an axial vector and a polar one, i.e., the electric field generated by point charges and the magnetization current density vector field, we have^38,39^
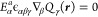
59from which it follows that
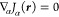
60

**Figure 1:**
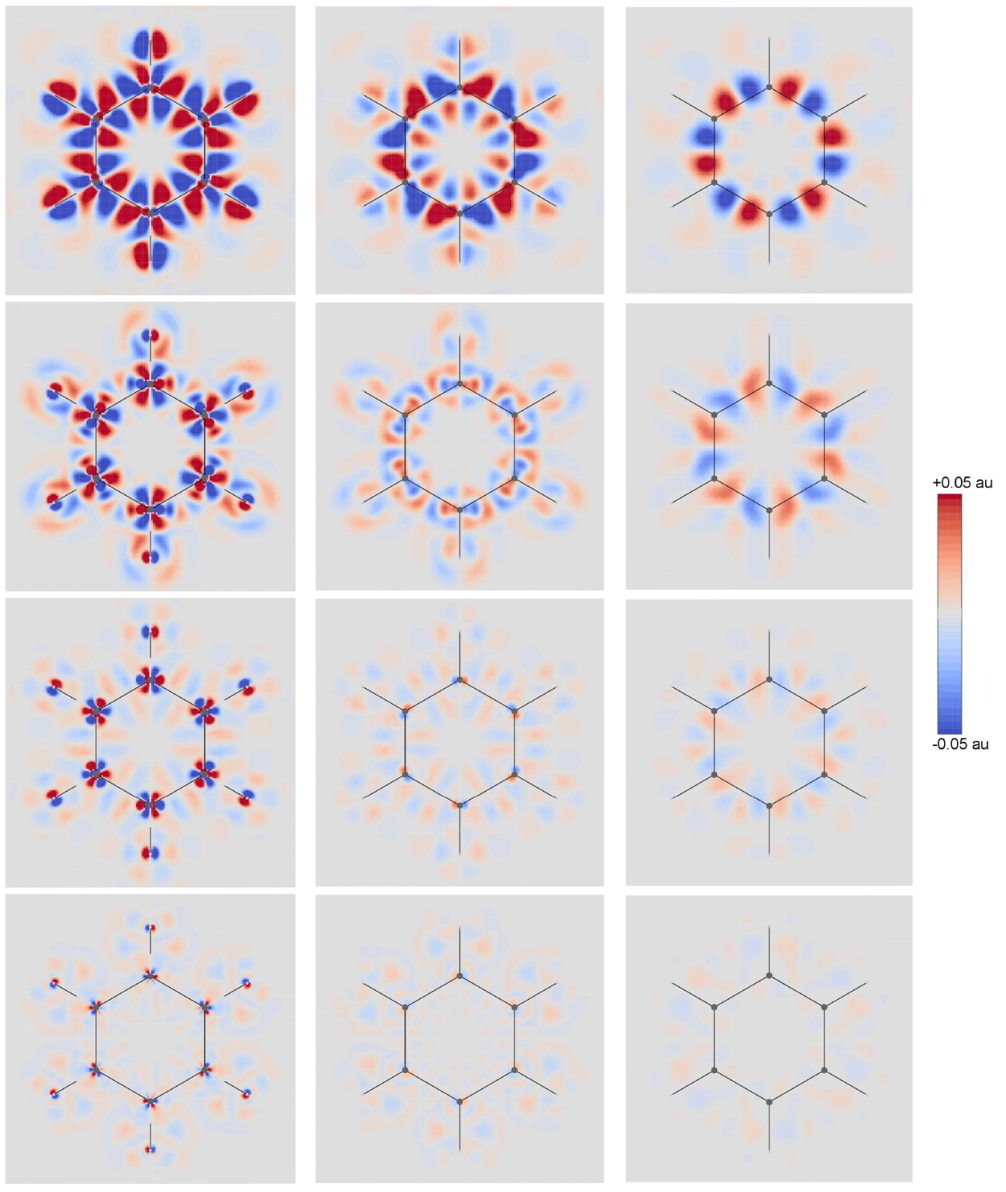
Diverging color map of ∇_α_*J*_α_ induced by a static magnetic field *B*_*z*_**ϵ**_3_ for the benzene molecule calculated on three different planes at BHandHLYP level of theory. The CPK (Corey-Pauling-Koltun) color scheme colors “atom” objects by the atom (element) type. From top to bottom, we have the four different basis sets adopted, i.e., aug-pcseg-0, aug-pcseg-1, aug-pcseg-2, and aug-pcseg-3 respectively. From left to right, we have the molecular plane, −0.5 and −1 au, respectively.

The condition ∇_α_*J*_α_(***r***) = 0 is fully satisfied only if the state functions are exact eigenfunctions of a model Hamiltonian and therefore satisfy the off-diagonal hypervirial theorem for the position operator, i.e., in HF, DFT, or Full-CI approaches^40,41^ as illustrated in Figure 1 for the divergence of first-order magnetically induced current density in the benzene molecule tending toward the complete basis set limit. This problem anyway, comes from the nonrelativistic current density vector, eq 30, because, as discussed before for spin contributions, see eqs 38 and 43, the charge-current conservation condition is satisfied for symmetry reasons related to the involved quantities, regardless electronic structure calculation method and the adopted basis set. Looking at the Larmor current induced by nuclear magnetic dipole moments, i.e. the last term of eq 48, it seems that a divergence of this current is always expected (also in the case of an exact calculation or in a complete basis set limit). That is not true because it can be shown that using Rayleigh–Schrödinger perturbation theory, at first order in the magnetic field given by nuclear magnetic dipoles, also a paramagnetic term can be obtained^2^
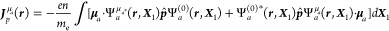
61with
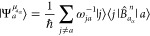
62
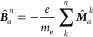
63
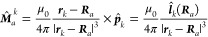
64

The sum of these paramagnetic and diamagnetic terms is gauge invariant and the integral conservation condition is satisfied in the case of a complete basis set and exact eigenfunctions of a model Hamiltonian.^2^
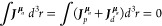
65

The same seems to be expected in a finite field calculation at all order in the applied magnetic field. It is not in the scope of this paper to show a plot of the paramagnetic contribution given by nuclear magnetic dipoles. The condition ∇_α_*J*_α_ = 0 is compatible with the true induced relativistic current density. To show this let us consider that the Dirac’s equation of an electron in an electromagnetic field reads^42^
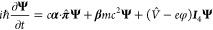
66with the 4-spinor **Ψ** defined as
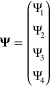
67and ***I***_4_ that is the 4 × 4 identity matrix. With reference to the Landau–Lifshitz approach used before, now it is possible to define
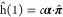
68and then to obtain using again the relation (14) and substituting ***A***(***r***) with eq 16
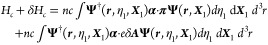
69From previous equation it follows that
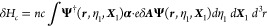
70

Using eq 11, the relativistic current density vector can be written as
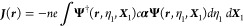
71and the satisfied charge-current conservation condition is in the case of static and uniform magnetic and electric fields^42^
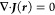
72

## 4 Implementation of Induced Spin Current Densities in a Linear Response Approach

The theoretical formulation of the total induced current density vector field can be straightly implemented within the UHF and UKS frameworks.^3,13,29^ The first term on the rhs of eq 44 describes the system response to an external applied magnetic field. In this context an explicit expression for the first term on the rhs of (44) can be provided according to the well-known equations for the first order-induced current density using a CTOCD approach^29,43−52^ or using London orbitals^12,53^ to avoid the origin dependence of the calculated current density vector field, being the same a subobservable according to a definition proposed by Hirschfelder.^54^ For the Zeeman current density, with the understanding that the reference frame is always chosen so that the quantization axis coincides with the direction of applied magnetic field, we can define^3^
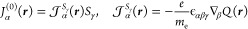
73

Similarly, for the spin–orbit coupling current, we have
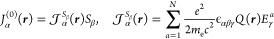
74with
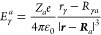
75and
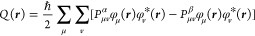
76where the density matrices are
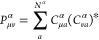
77
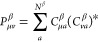
78defined using the Szabo and Ostlund notation.^55^ Orbital coefficients can be obtained from a Gaussian^56^ calculation or generated by the SYSMOIC suite of programs.^29^ The full procedure for the calculation of total induced current density vector field has been implemented in the freely available SYSMOIC program package.^29^ The implementation here reported seems to be easily extendable also to nonperturbative approaches^24,57^ when a magnetic field is applied only in the *z* direction in which the electron spin Zeeman Hamiltonian can be expressed as a scalar quantity, but this is not true for the one-electron spin–orbit coupling Hamiltonian.^25^ For the Larmor contribution given by the nuclear magnetic dipole **μ**_*a*_
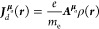
79the implementation at first order is straightforward, being
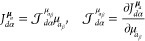
80and
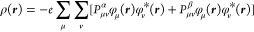
81

Note that **μ**_*a*_ is a vector with components equal to the maximum *z*-component expectation value of the magnetic dipole moment, for a given nucleus, in units of nuclear magnetons.

## 5 Calculation Details

Being in the case of spin currents the continuity equation satisfied for symmetry reasons, we have computed only the divergence of non relativistic current density, eq 30, calculated at first order in the applied magnetic field for the benzene molecule, prototypical of aromatic behavior for a magnetic field applied in the *z* direction. Thanks to its small size, very accurate computations have been carried out, using the BHandHLYP functional,^58^ recently shown to perform quite well,^41^ adopting basis sets of contracted functions which include terms of high angular momentum, taken from BSE. In particular, the aug-pcseg-X basis set series (with X = 0, 1, 2, 3) has been adopted.^59^ BHandHLYP perturbed coefficients for ***r*** ×  and  operators have been computed using the Gaussian 16 program package.^56^ Geometry was first optimized using the same functional with aug-cc-pVTZ basis set. In order to show some maps of induced spin currents, the CH_3_ radical in a doublet state and an open-shell graphene like system, i.e., the superoctazethrene^60^ have been taken into account. The geometries have been optimized with an unrestricted B3LYP calculation adopting the 6-31G(d) basis set. The keyword guess = mix has been used in the case of superoctazethrene to account for the singlet open-shell character. Then single point calculations made with the same functional and a 6-311+G(2d,p) basis set adopting the 6d 10f keywords have been performed to obtain the unperturbed coefficients needed for the evaluation of spin density and spin currents using the SYSMOIC program facilities.^29^ All the electronic structure calculations have been performed using the Gaussian 16 program package.^56^

## 6 Results and Discussion

**Figure 2:**
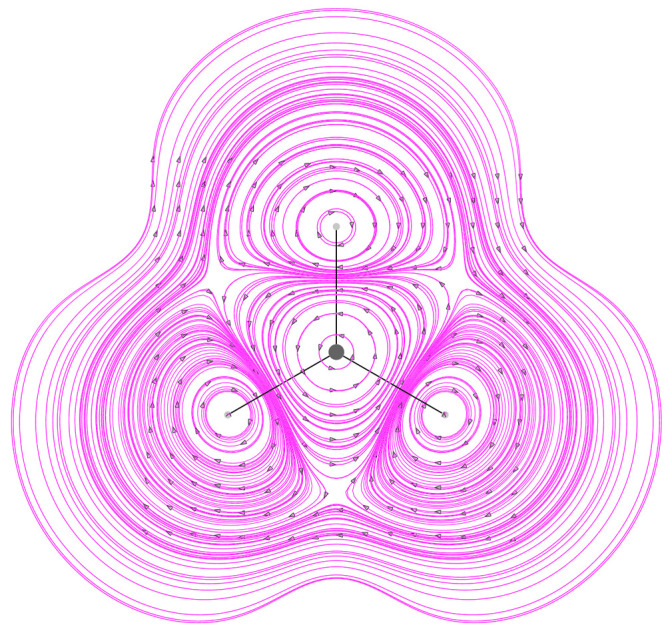
Magnetization current density vector field, eq 38, induced on the molecular plane in the CH_3_ radical by a static magnetic field *B*_*z*_**ϵ**_3_ considered to be coincident with the quantization axis represented with a streamlines map.

**Figure 3:**
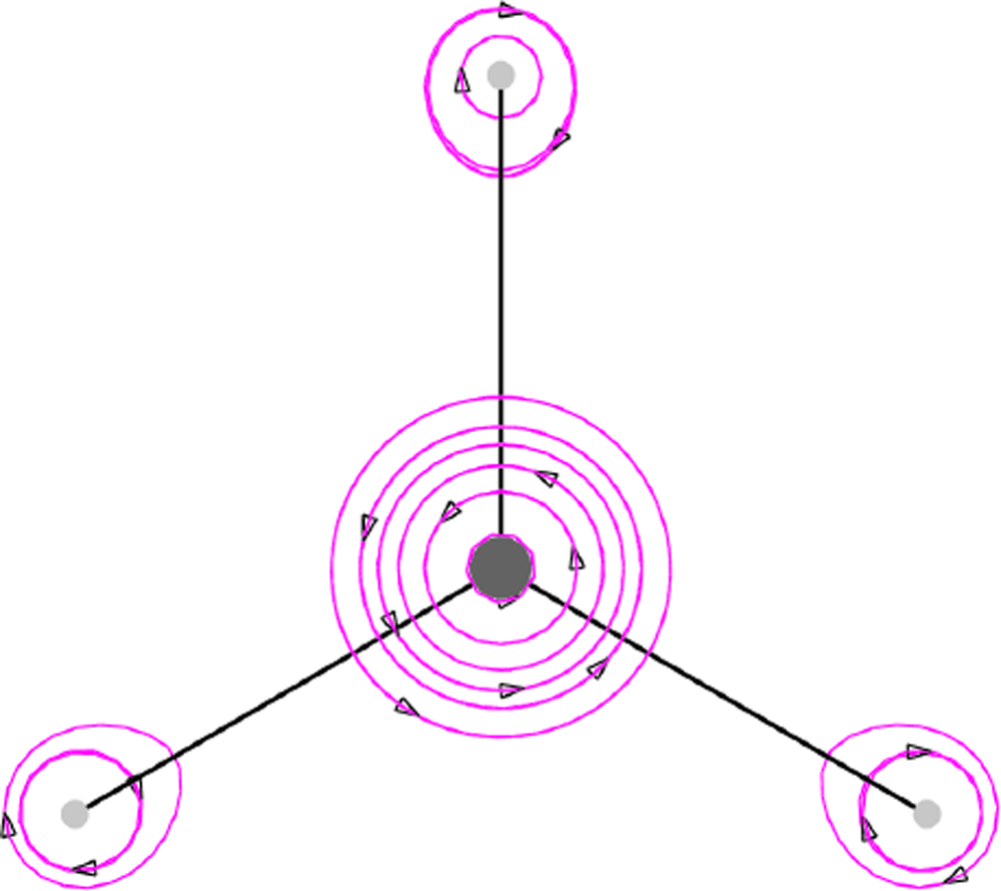
Spin orbit coupling current density vector field, eq 43, on the molecular plane induced in the CH_3_ radical by a static magnetic field *B*_*z*_**ϵ**_3_ considered to be coincident with the quantization axis represented with a streamlines map. Note that to produce this map, in the implementation done in atomic units, the factor  has been not taken into account.

**Figure 4:**
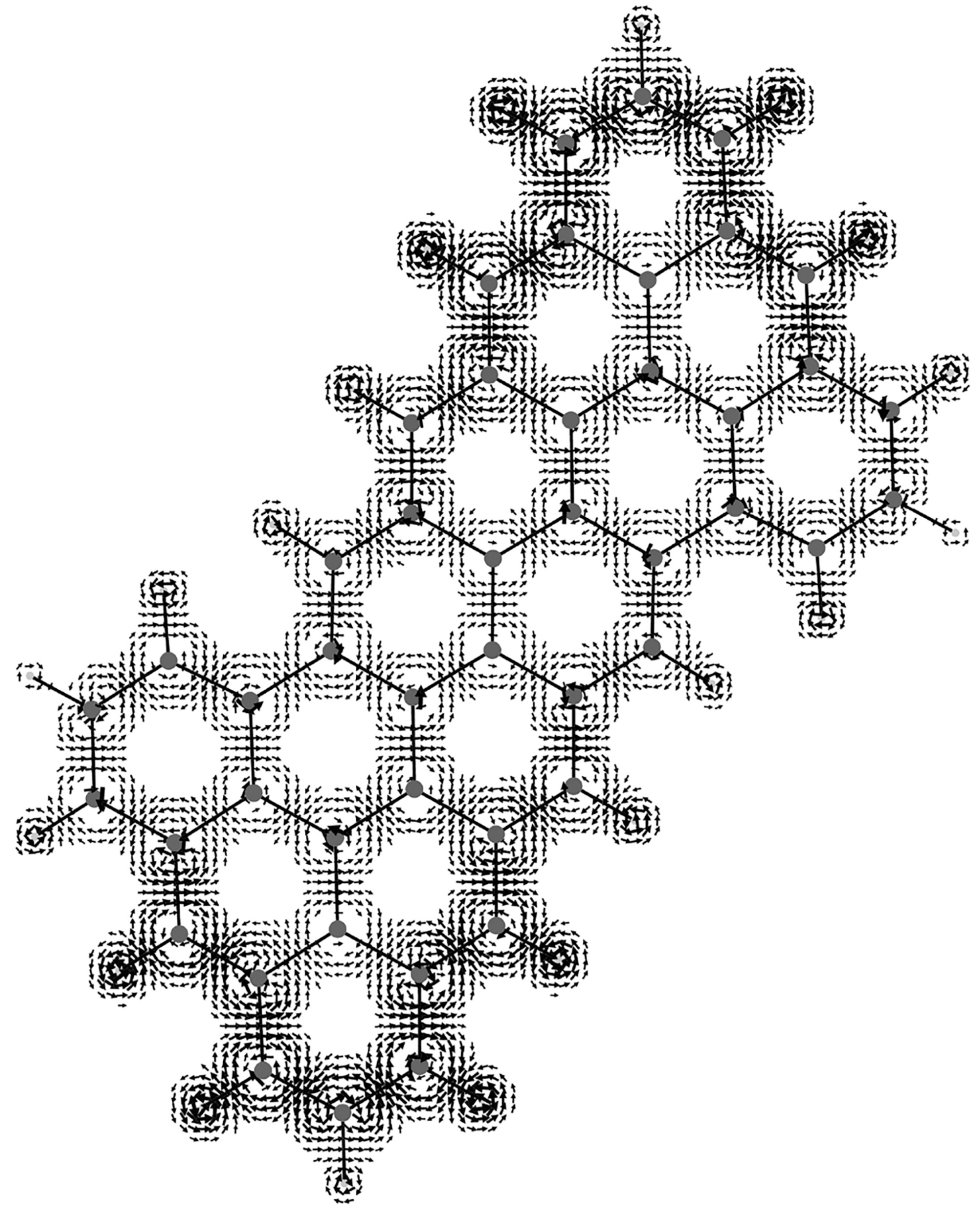
Magnetization current density vector field, eq 38, on the molecular plane induced in the superoctazethrene molecule by a static magnetic field *B*_*z*_**ϵ**_3_ considered to be coincident with the quantization axis.

**Figure 5:**
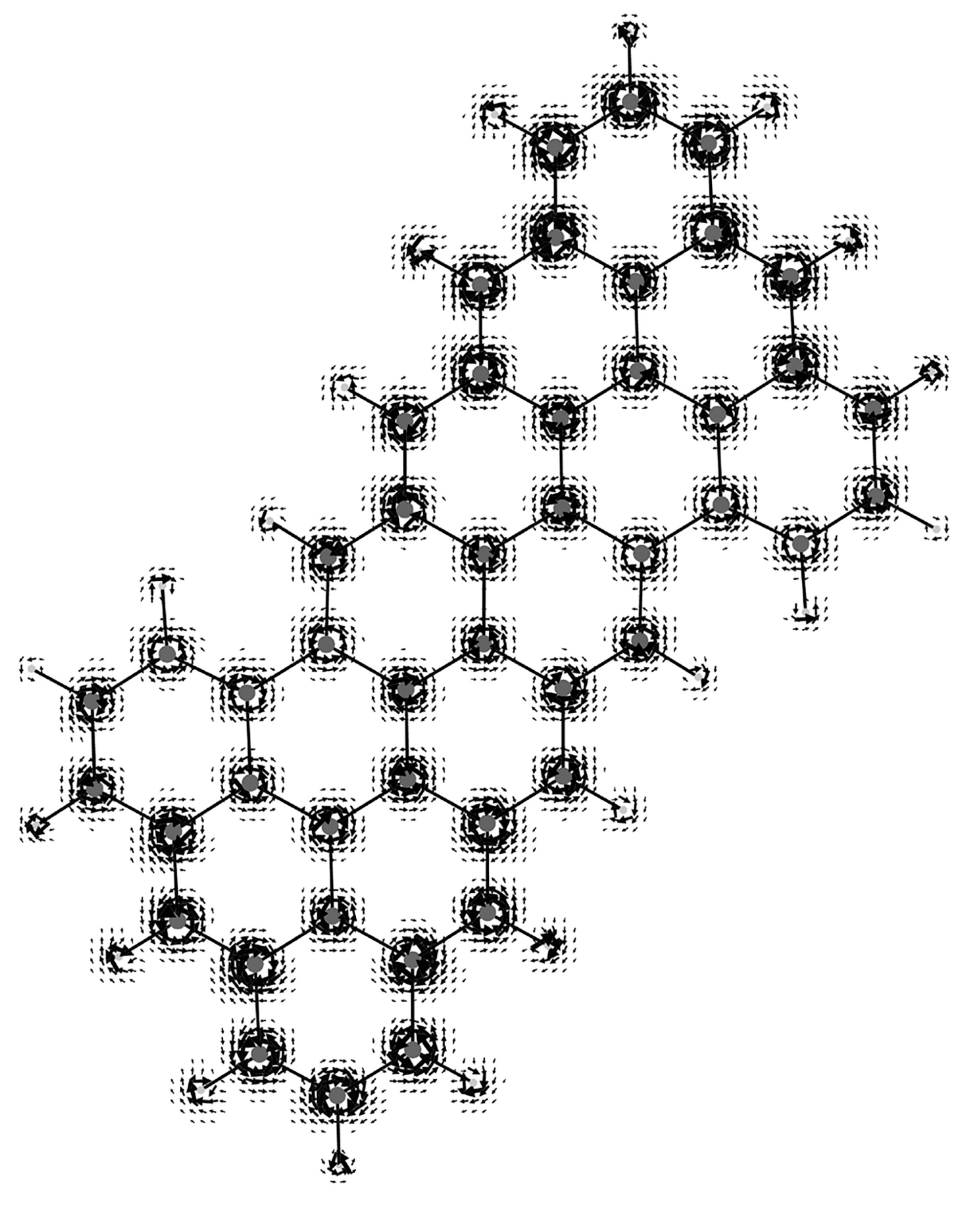
Spin orbit coupling current density vector field, eq 43, on the molecular plane induced in the superoctazethrene molecule by a static magnetic field *B*_*z*_**ϵ**_3_ considered to be coincident with the quantization axis. Note that to produce this map, in the implementation done in atomic units, the factor  has been not taken into account.

**Figure 6:**
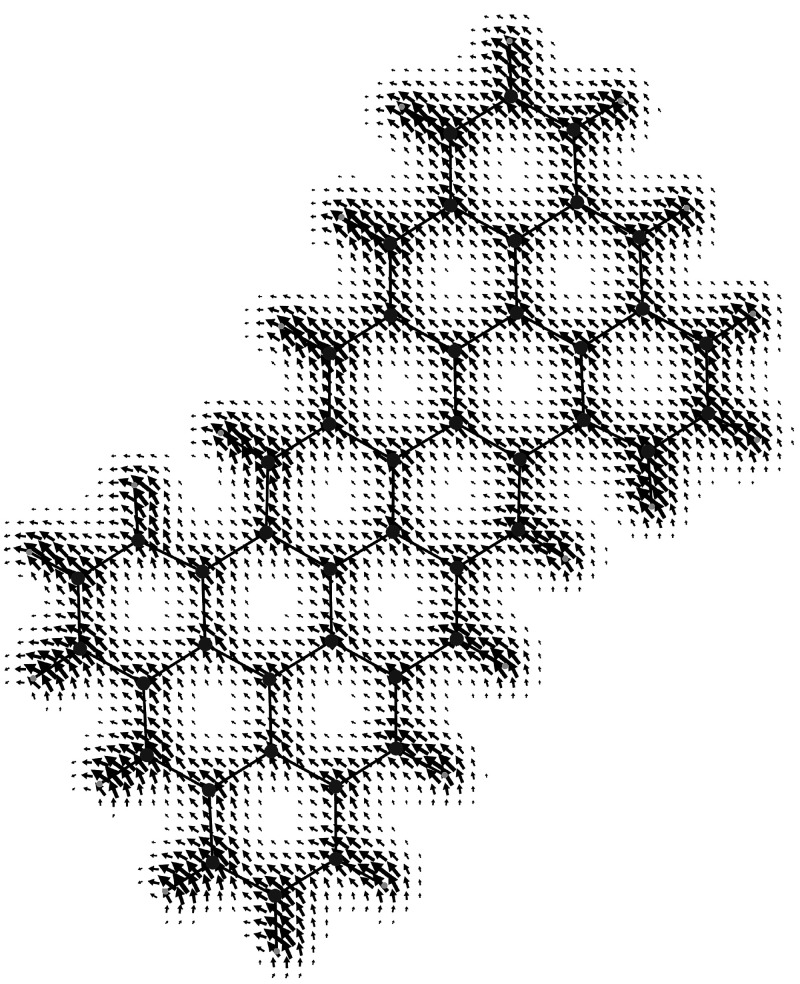
Larmor current density vector field, given by nuclear magnetic dipoles, at −1 a.u. on the molecular plane induced in the superoctazethrene molecule by a static magnetic field *B*_*z*_**ϵ**_3_. Note that to produce this map, in the implementation done in atomic units, the ratio  has been not taken into account.

**Figure 7:**
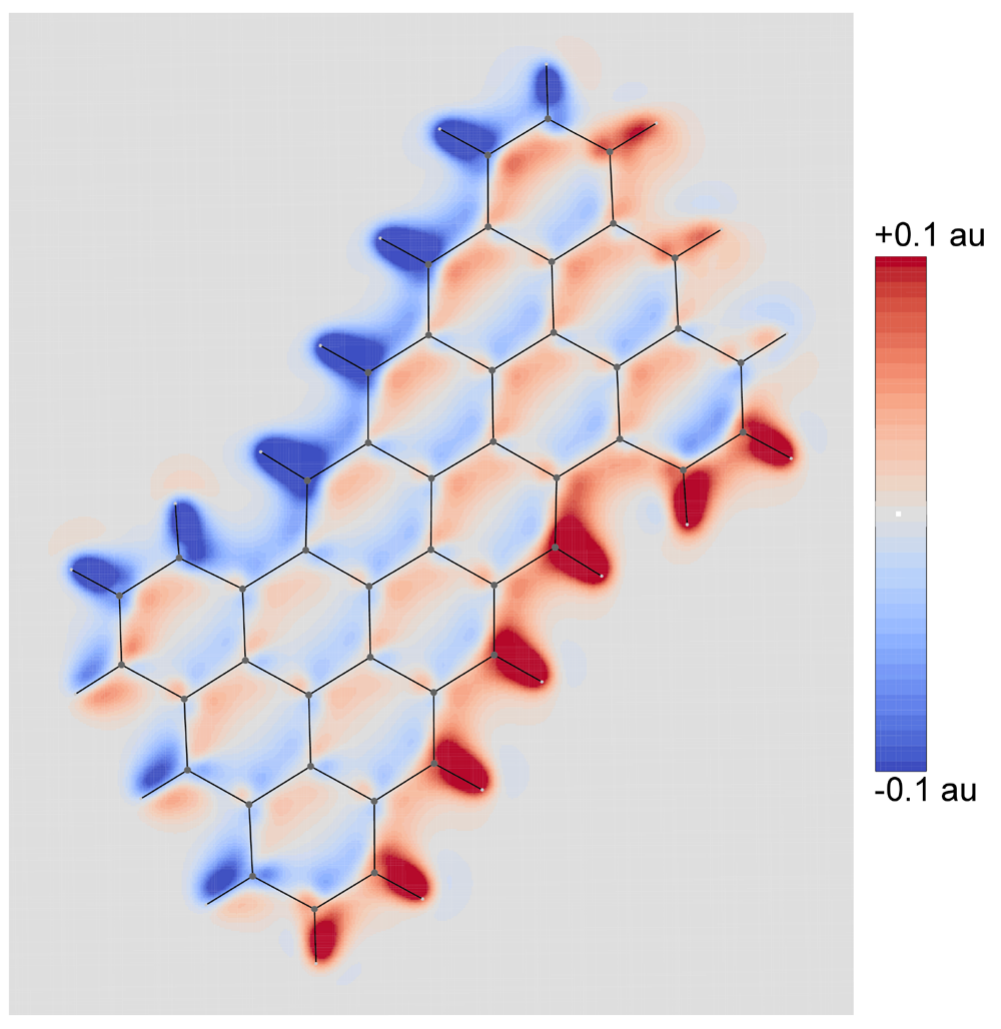
Divergence of Larmor current density vector field, given by nuclear magnetic dipoles, at −1 a.u. on the molecular plane induced in the superoctazethrene molecule by a static magnetic field *B*_*z*_**ϵ**_3_. Note that to produce this map, in the implementation done in atomic units, the ratio  has been not taken into account.

Diverging color maps of ∇_α_*J*_α_ of first-order induced current density are illustrated in Figure 1 for a magnetic field applied along the *z* direction in the benzene molecule using a CTOCD-DZ2 approach.^47^ As we can observe, an increase in the basis set size reduces the divergence of the current density vector field in all directions. Four maps of induced spin currents for a magnetic field applied along the *z*-axis (that is considered as the quantization axis) on the molecular plane are illustrated in Figures 2–5 for the CH_3_ radical and superoctazethrene molecules in the D_3*h*_ and C_2*h*_ symmetry point groups respectively for the electron Zeeman and the spin orbit coupling currents. From these maps it follows that the topological structure of the two kind of spin currents is not so different. A difference can be seen near the nuclear positions where a divergence due to the point charge character of the electric field is expected for the spin–orbit coupling current, as well as a current cusp. Another difference is the fast decay of the spin orbit coupling current, compared to the spin magnetization current, observed moving away from nuclear positions due to the electric field dependence as . A plot of the total spin current is not useful due to the different magnitude of these currents not taken into account in the maps here shown being the spin–orbit coupling current smaller than the spin magnetization current roughly by a factor . Looking at Figures 4 and 5 different tropicities can be observed in both maps together with a clear evidence of the regions in space where unpaired electrons have a higher probability to be found. It is clear by inspection of equations and maps that spin–orbit coupling (SOC) currents enhance the curvature and give rise to a previously unobserved current cusps according to the fully 4-components relativistic calculation as seen in ref (27). A plot of the Larmor contributions given by the nuclear magnetic dipoles is also presented in Figure 6 together with its divergence in Figure 7.

## 7 Conclusions

In the present manuscript it has been illustrated that the introduction of a one-electron spin–orbit coupling contribution in the approximate total induced current density vector field, as defined in eq 44, does not change the charge-current conservation condition expected to be fulfilled (***∇***·***J*** = 0) in the case of applied static and uniform magnetic and electric fields for an exact calculation or in the limit of a complete basis set. The divergence comes to be zero for the spin magnetization current eq 38 due to the presence of the curl. For the spin–orbit coupling current, eq 43, instead, the divergence is zero thanks to the scalar product between an axial vector and a polar one as discussed before.^38,39^ To illustrate that the divergence of the total induced current density vector field is not zero in approximate calculations some maps of this scalar quantity have been shown in a linear response approach using a CTOCD-DZ2 method to avoid the origin-dependence of first order induced current density. The condition ∇_α_*J*_α_ = 0 is fully satisfied only if the state functions are exact eigenfunctions of a model Hamiltonian and therefore satisfy the off-diagonal hypervirial theorem for the position operator, i.e., in HF, DFT or Full-CI approaches.^40,41^ Some maps of spin currents have been illustrated for the CH_3_ radical and the superoctazethrene^60^ molecules. A contribution coming from the two-electron spin–orbit coupling interaction is also expected, as discussed in the main text, in the definition of the total many body induced current density vector field, due to the presence of the vector potential, but it is expected to be smaller than the others like the spin magnetization current, the nonrelativistic and the one-electron spin orbit coupling ones, being in heavy elements, the one-electron term of the spin–orbit coupling Hamiltonian the dominating part.^61,62^ Furthermore, neglecting the two-electron spin–orbit coupling contribution it is possible to implement currents, as defined in eq 44, in standard nonrelativistic UHF and UDFT calculations in a linear approach fashion or in a nonrelativistic current density functional theory picture.^23,24^ For the reasons here exposed and that the features of a full 4-components relativistic calculation are observed, it seems that the equations here reported can be applied to molecular systems where the strictly central field condition is lost. A plot of the Larmor contribution given by nuclear magnetic dipoles is also shown together with a map of its divergence different from zero also in a complete basis set limit. This divergence is erased by the divergence of the paramagnetic contribution as shown in terms of the integral conservation condition eq 65.

## Dedication

This paper is dedicated to Professor Paolo Lazzeretti on the occasion of his 80th birthday.
